# Rush Medical College

**Published:** 1856-12

**Authors:** 


### EDITORIAL.

*Rush Medical College.*

The regular term of lectures in this Institution commenced on the first Monday in last month. The general introductory lecture was delivered by Prof. H. A. Johnson, and was listened to with much pleasure by the audience. The lecture was beautifully written, and in sentiment strictly appropriate to the occasion. The number in attendance during the present College term does not vary materially from that of former years; and the advantages they possess cannot be easily excelled. Aside from the regular courses of instruction in the College, Members of the Class have access to the Marine and Mercy Hospitals, both of which are well filled with patients. The Professor of Surgery gives clinical instruction four mornings each week in the Marine Hospital, and the Professor of Practical Medicine six in the Mercy Hospital. By this arrangement every student, taking the Hospital tickets, can receive instruction directly at the bed-side, at least four mornings each week, and yet have the number in attendance at any one time so limited as to permit the most full and careful examination of each individual case, including the frequent practice by the student of auscultation and percussion.

